# Genome editing using a versatile vector-based CRISPR/Cas9 system in *Fusarium* species

**DOI:** 10.1038/s41598-022-20697-4

**Published:** 2022-09-28

**Authors:** Sota Shinkado, Hiroki Saito, Masaya Yamazaki, Shunsuke Kotera, Takayuki Arazoe, Tsutomu Arie, Takashi Kamakura

**Affiliations:** 1 Faculty of Science and Technology, Tokyo University of Science, 2641 Yamazaki, Noda, Chiba 278-8510 Japan; 2 United Graduate School of Agricultural Science, Tokyo University of Agriculture and Technology (TUAT), 3-5-8 Saiwai-cho, Fuchu, Tokyo 183-0054 Japan; 3 Faculty of Agriculture, Institute of Agriculture, Tokyo University of Agriculture and Technology, 3-5-8 Saiwai-cho, Fuchu, Tokyo 183-0054 Japan; 4 Present Address: Graduate School of Biological Science, Nara Institute of Science and Technology, 8916-5 Takayama-cho, Ikoma, Nara 630-0192 Japan

**Keywords:** Fungi, Double-strand DNA breaks, Homologous recombination, Functional genomics

## Abstract

*Fusarium* species include important filamentous fungal pathogens that can infect plants, animals, and humans. Meanwhile, some nonpathogenic *Fusarium* species are promising biocontrol agents against plant pathogens. Here, we developed a genome editing technology using a vector-based CRISPR/Cas9 system for *Fusarium oxysporum* f. sp. *lycopersici* (*Fol*). This optimized CRISPR/Cas9 system, harboring an endogenous U6 small nuclear RNA promoter for the expression of single-guide RNA and an endogenous H2B nuclear localization signal for the localization of Cas9, enabled efficient targeted gene knock-out, including in the accessory chromosomal regions in *Fol*. We further demonstrated single crossover-mediated targeted base editing and endogenous gene tagging. This system was also applicable for genome editing in *F. oxysporum* f. sp. *spinaciae* and *F. commune* without any modifications, suggesting that this CRISPR/Cas9 vector has a potential application for a broad range of researches on other *Fusarium* species.

## Introduction

The genus *Fusarium* belongs to ascomycetes, and the genus includes over 1500 species^1^. *Fusarium* species are widely distributed in the environment, and its several strains are capable of causing serious diseases in plants, animals, and humans^1^. *F. oxysporum*, a species of the genus *Fusarium*, is also widely distributed in various environments, including the phytosphere and rhizosphere, and the species again includes strains that are plant, human, and animal pathogens and many nonpathogens. Plant pathogenic *F. oxysporum* is famous as a soilborne pathogen that infects host plants via the roots. The pathogen eventually colonizes the vascular tissues and causes systemic yellowing, wilting, and death in plants by blocking the vessel translocation of water and nutrients^1^. Although *F. oxysporum* causes diseases in various plants, individual strains show selective pathogenicity and are classified into forms (*formae speciales*, f. sp.) based on their host species^2^. For example, *F. oxysporum* isolates responsible for tomato (*Solanum lycopersicum*) wilt belong to the f. sp. *lycopersici*; similarly, those causing wilting on spinach (*Spinacia oleracea*) belong to the f. sp. *spinaceae*. These pathogenic *F. oxysporum* strains carry accessory chromosomes (ACs), which are nonessential for their growth in contrast to the core chromosomes^3^. ACs harbor lineage- or strain-specific genomic and structural compositions, such as unique genes, different codon usage biases, and rich transposable elements (TEs)^3^. In addition, ACs have many pathogenicity-related factors, and are directly linked to host-specific pathogenicity in some plant pathogenic *F. oxysporum*^4–7^. ACs of pathogenic strains are capable of being horizontally transferred to nonpathogenic strains, which lead the nonpathogenic strains to gain pathogenicity. However, little is known about the molecular mechanisms of host specificity, and functional analysis of these genes in ACs is often hampered by TE-enriched traits.

Nonpathogenic *Fusarium* strains are often isolated from asymptomatic crop plants and are capable of colonizing plant roots without inducing any disease symptoms. Recently, a novel technique to control Bakanae disease, caused by *F. fujikuroi*, using the nonpathogenic *F. commune* isolate W5 was reported^8^. Spray treatment with W5 reduced pathogen invasion of rice flowers, and W5 was transmitted to the next plant generation via seeds. However, the detailed molecular mechanisms of the biocontrol capacity of the nonpathogenic fungus have not been elucidated. Efficient and versatile genome manipulation tools would facilitate the study of the capacity of nonpathogenic biological agents.

Clustered regularly interspaced short palindromic repeats (CRISPR)/ CRISPR-associated protein (Cas) systems have revolutionized RNA-guided genome editing technology^9^. The type II CRISPR/Cas9 system from *Streptococcus pyogenes* (SpCas9) is widely used as a genome editing tool in various organisms^10–12^. The single-guide (sg) RNA/Cas9 ribonucleoprotein (RNP) complex can introduce a site-specific DNA double-strand break (DSB) at the hybridizing locus of the sgRNA/target DNA. In the case of SpCas9, protospacer-adjacent motif (PAM), the 5′-NGG-3′ motif, is required for effective cleavage of the target site. DSBs are repaired by two competing pathways in most filamentous fungal cells: non-homologous end joining (NHEJ) repair and homologous recombination (HR) repair. NHEJ-mediated genome editing can introduce insertions and/or deletions (indels) at the target site without donor DNAs; however, the mutation type cannot be controlled, and large deletions often occur during DNA repair in the absence of donor sequences in the filamentous fungal genome^13,14^. In contrast, HR repair can be induced by DNA cleavages in many filamentous fungi, which enables to introduce precise mutations, such as targeted gene knock-out, knock-in, and replacement^14^. In the rice blast fungus *Pyricularia oryzae* (*Magnaporthe oryzae*), site-specific DSB could induce single crossover-type HR, which occurs DNA strand exchanges between the cleaved DNA and donor plasmid DNA. Single crossover-mediated genome editing enabled efficient targeted gene disruption, base editing, and endogenous gene tagging^15^, but the feasibility of this strategy in other fungi has not been validated. Genome editing using the CRISPR/Cas9 system has rapidly developed in filamentous fungi, including *Fusarium* species^14,16^. In *F. oxysporum* f. sp. *vasinfectum* (*Fov*), which causes destructive diseases in cotton, an in vitro-assembled Cas9 RNP complex was used as a genome editing tool, and the maximum efficiency of targeted gene disruption and endogenous gene tagging was elevated up to 50%^17,18^. However, efficient genome editing in the AC regions has not been validated. To the best of our knowledge, CRISPR/Cas9-based genome editing has been applied to *F. solani* var. *petroliphilum*^19^, *F. proliferatum*^20^, *F. venenatum*^21^, and *F. graminearum*^22^. In *F. solani* and *F. proliferarum*, an in vitro-assembled Cas9 RNP complex has also been used for genome editing. In *F. graminearum*, a vector-based CRISPR/Cas9 system was used for genome editing, but its efficiency was very low without counter selection. In *F. venenatum*, an AMA1-based self-replication plasmid was used for the transient expression of the CRISPR/Cas9 system, but this system and strategy can be used for limited fungal species. In this study, we developed a vector-based CRISPR/Cas9 system and demonstrated highly efficient targeted gene knock-out, knock-in, and replacement, including in AC regions in *F. oxysporum* f. sp. *lycopersici* (*Fol*). The developed system was also applied to genome editing in *F. oxysporum* f. sp. *spinaciae* (*Fos*) and *F. commune* without any modifications to the CRISPR/Cas9 vector*.*

## Results

### Development of optimized CRISPR/Cas9 vector and HR-mediated targeted gene knock-out

**Figure 1:**
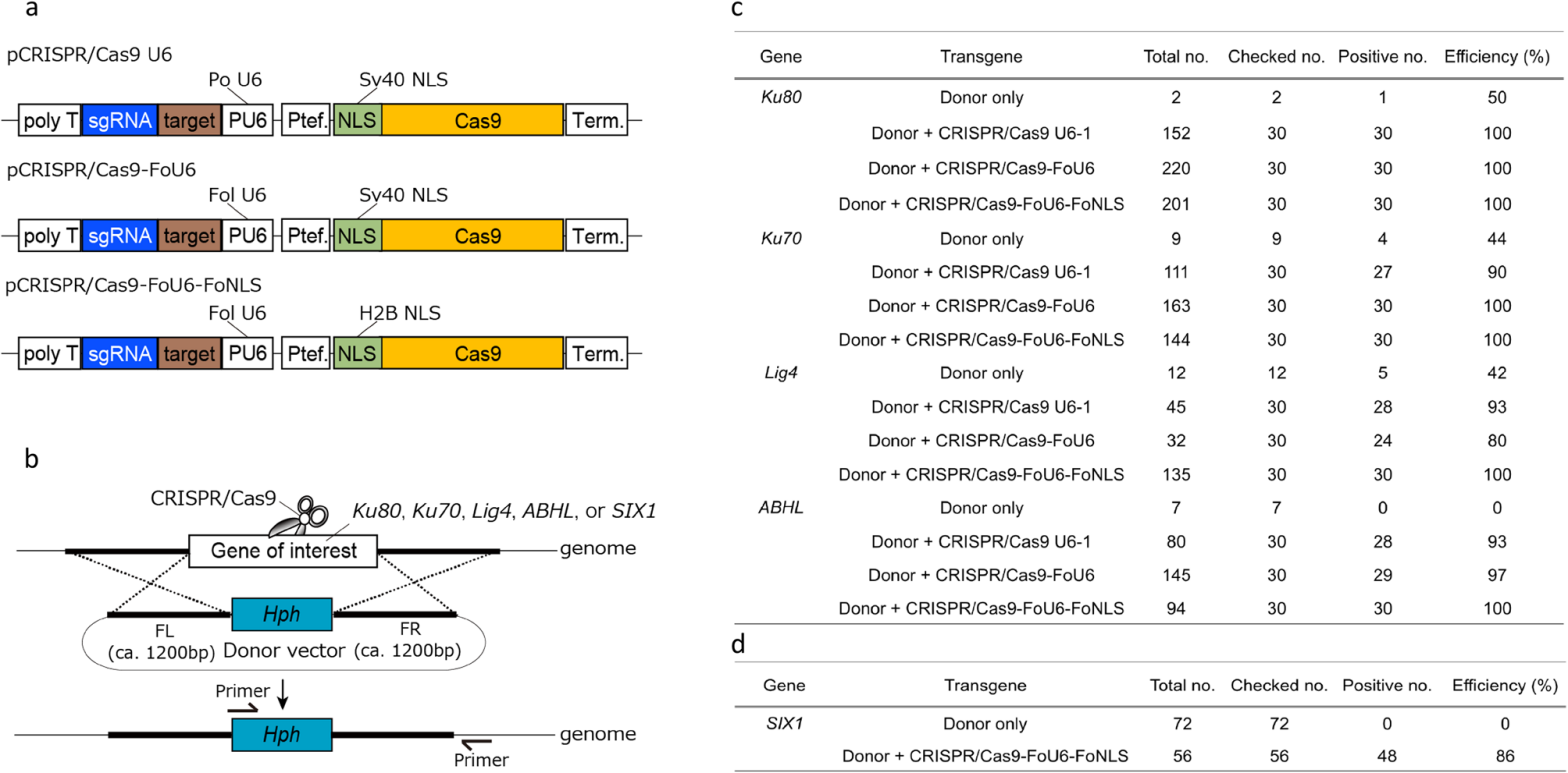
Homologous recombination (HR)-mediated targeted gene knock-out using optimized CRISPR/Cas9 system in *Fusarium oxysporum* f. sp. *lycopersici* (*Fol*). (**a**) Schematic representation of CRISPR/Cas9 systems used in this study. Poly T: five-thymine repeat as a terminator of the U6 promoter, PU6: U6 promoter, Ptef: translation elongation factor promoter, NLS: nuclear localization signal, Term.: terminator, Po U6: U6 promoter derived from *Pyricularia oryzae*, Fol U6: U6 promoter derived from *Fol*, Sv40 NLS: nuclear localization signal derived from Simian virus 40, and H2B NLS: histone H2B nuclear localization signal derived from *Fol*. (**b**) Schematic representation of HR-mediated targeted gene knock-out with CRISPR/Cas9 system. FL: flanking region of left side, FR: flanking region of right side, *Hph*: hygromycinB phosphotransferase gene cassette. (**c**) Knock-out efficiency of *Ku80*, *Ku70*, *Lig4*, and bacterial alpha/beta hydrolase-like protein (*ABHL*) using pCRISPR/Cas9 U6-1, pCRISPR/Cas9-FoU6 and pCRISPR/Cas9-FoU6-FoNLS. (**d**) Knock-out efficiency of *SIX1* using pCRISPR/Cas9-FoU6-FoNLS.

We previously established a vector-based CRISPR/Cas9 system for the rice blast fungus *P. oryzae* (pCRISPR/Cas9 U6-1) (Fig. 1a)^23^. To optimize this system for *Fol*, an endogenous U6 promoter was isolated and exchanged for that of *P. oryzae* on the vector (Fig. 1a; pCRISPR/Cas9-FoU6). Further, a nuclear localization signal derived from Simian virus 40 (Sv40 NLS) at the N-terminus of Cas9 was replaced by the endogenous histone H2B NLS (H2B NLS) in *Fol* (pCRISPR/Cas9-FoU6-FoNLS) (Fig. 1a), because Sv40 NLS was not suitable for translocation of Cas9 into the nucleus in some fungi^17,24^. The nuclease activity of these CRISPR/Cas9 vectors were evaluated by the efficiency of HR-mediated targeted gene knock-out of the *Ku80*, *Ku70*, and *Lig4* genes (Fig. 1b), which are involved in NHEJ-mediated repair^25,26^. CRISPR/Cas9 and donor vectors targeting each gene were simultaneously introduced into the protoplasts. The number of hygromycin B-resistant colonies was dramatically increased by co-introduction of CRISPR/Cas9 and donor vectors, compared with that of only donor vectors (Fig. 1c). PCR analysis showed that the use of the CRISPR/Cas9 vectors including CRIPSR/Cas U6-1 for *P. oryzae* increased the knock-out efficiency of *Ku80* (100%), *Ku70* (90–100%), and *Lig4* (80–100%) (Fig. 1c). Using CRISPR/Cas9-FoU6-FoNLS, knock-out efficiencies were elevated up to 100% for all the three genes (Fig. 1c), but the introduction with only donor vectors also showed high disruption efficiencies (42–50%) in these experiments. Thus, we further evaluated the system by disrupting the bacterial alpha/beta hydrolase-like protein (*ABHL*) gene, which is located in the AC region (contig14) of the *Fol* isolate 4287 genome. In the case of *ABHL*, 1000 bp homology arms were used for the disruption. The knock-out efficiency of *ABHL* using the CRISPR/Cas9 systems were 93–100%, respectively (Fig. 1c). The use of CRISPR/Cas9-FoU6-FoNLS also exhibited the highest knock-out efficiency (100%). From these results, we used pCRISPR-FoU6-FoNLS for further analysis. To evaluate the feasibility of this system, *SIX1*, located in the AC region (contig14), was selected as the target gene. In the case of *SIX1*, the homology arms (FL: 706 bp; FR: 896 bp) were used for the deletion of a part of *SIX1* (Fig. S1), because many TEs and repetitive elements are located around the gene. The knock-out of *SIX1* using CRISPR/Cas9 system also showed high efficiency (86%) (Figs. 1d, S2). These results demonstrated that the optimized CRISPR/Cas9 system enabled the effective introduction of DSBs into the targeted genes and was applicable for efficient genome editing, including in the AC regions in *Fol*.

**Figure 2:**
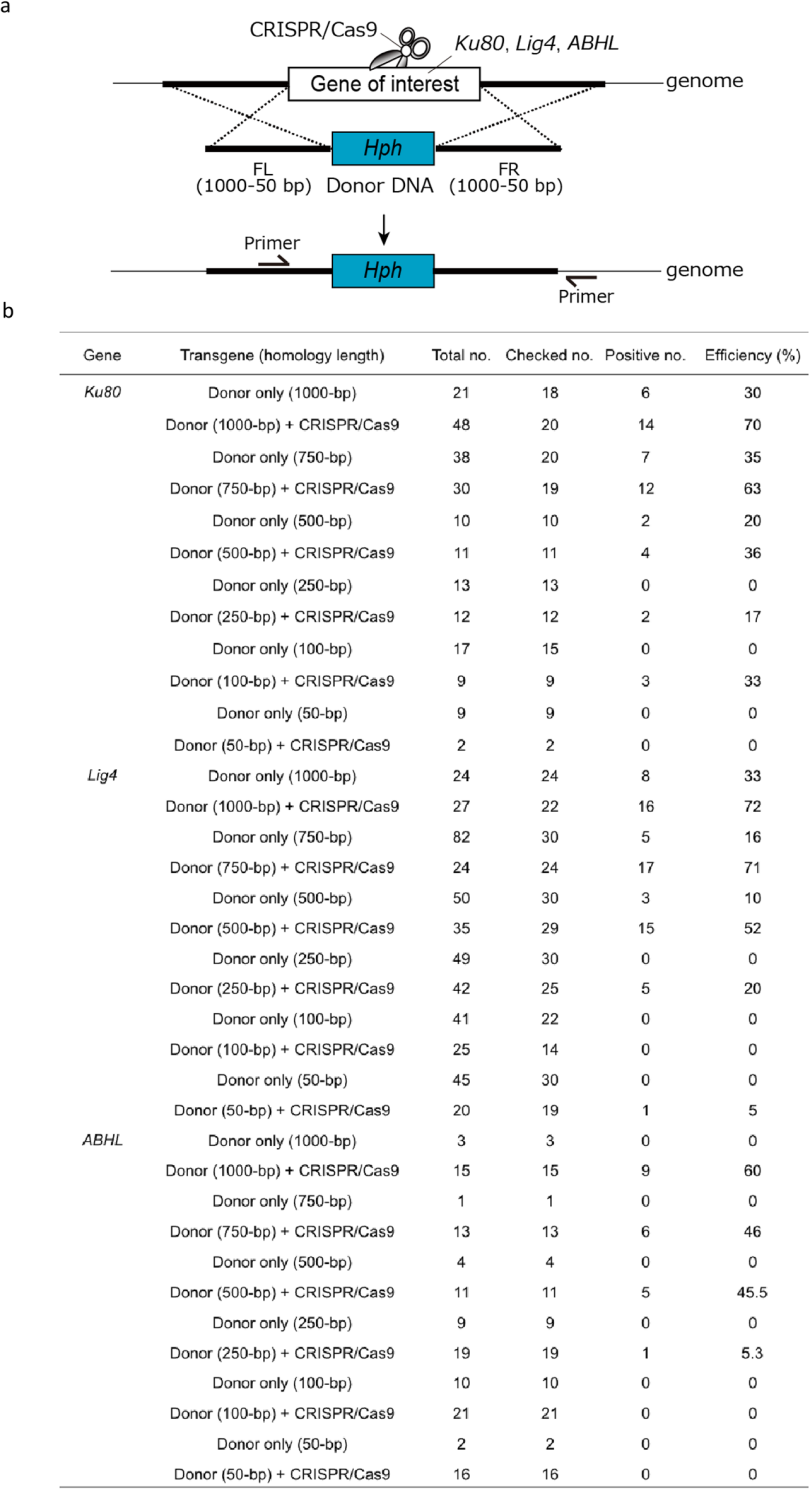
Short homology arm-mediated targeted gene disruption using CRISPR/Cas9. (**a**) Schematic representation of HR-mediated targeted gene knock-out using donor DNA fragments with short homology arms. (**b**) The efficiencies of HR-mediated targeted *Ku80, Lig4,* and *ABHL* knock-outs using donor DNA fragments with short homology arms.

Although HR-mediated genome editing was effective in *Fol*, the acquisition of homology arms of sufficient length is often prevented by existing TEs and/or repetitive elements. Thus, we attempted to develop a short homology arm-mediated gene disruption strategy. We truncated the homology arms (1000, 750, 500, 250, 100, or 50 bp) of the donor vector by PCR (Fig. 2a). The amplified fragments and CRISPR/Cas9 vector targeting *Ku80*, *Lig4, or ABHL* were co-introduced into the protoplasts. PCR analysis showed that the shortened fragment type donor DNA could be used for targeted gene disruption, although the number of transformants and disruption efficiencies tended to depend on the length of the homology arms and the target gene locus (Fig. 2b).

### Single crossover-mediated targeted base editing and endogenous gene tagging

**Figure 3:**
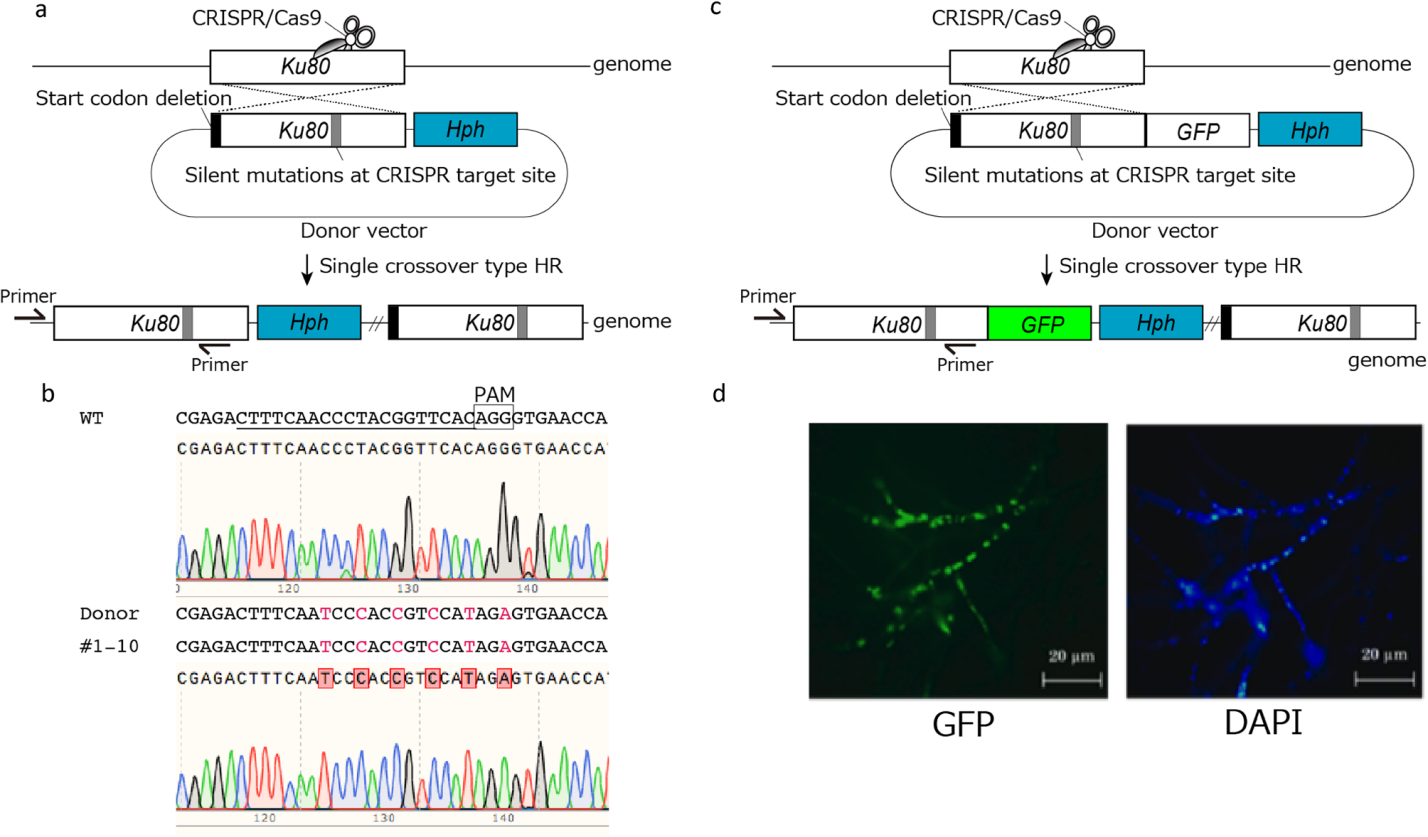
Single crossover-mediated targeted base editing and endogenous gene tagging using CRISPR/Cas9. (**a**) Schematic representation of the single crossover-mediated *Ku80* base editing. Silent mutations were introduced into the CRISPR/Cas9 target site of *Ku80* homologous sequence to evade CRISPR/Cas9 cleavage. (**b**) Sequences around the CRISPR/Cas9 target site in the wild type, donor vector, and transformants #1–10. The chromas data obtained by sequencing analysis of WT and transformant #1 were shown under the sequences. (**c**) Schematic representation of single crossover-mediated *Ku80* gene tagging with the *GFP* reporter gene. The stop codon was removed from the *Ku80* homologous sequence, and the *GFP* gene was fused at the C-terminus. (**d**) GFP fluorescence in the GFP-tagged transformants. The bars are 20 µm.

We previously succeeded in single crossover-mediated targeted base editing and endogenous gene tagging in *P. oryzae*^15^. To evaluate this strategy in *Fol*, the *Ku80* region containing point mutations (silent mutations) at the CRISPR/Cas9 target site was obtained as a single homology arm (Fig. 3a). CRISPR/Cas9 and donor vectors were simultaneously introduced into the protoplasts, and 24 independent hygromycin B-resistant colonies were obtained. Ten randomly picked up colonies were sequenced around the CRISPR target site, and the analysis showed that all the colonies (100%) contained the desired mutations at the target site (Fig. 3b and Fig. S3). To investigate whether the single crossover-type HR can also be exploited for endogenous gene tagging in *Fol*, GFP-fused *Ku80* with silent mutations was used for the homology arm (Fig. 3c). All 10 of the randomly selected independent hygromycin B-resistant colonies contained the desired mutations at the target site (100%) (Fig. S3) and showed nuclear-localized GFP fluorescence (Fig. 3d). These results indicated that the CRISPR/Cas9-mediated DSBs enabled efficient single crossover-mediated targeted base editing and endogenous gene tagging in *Fol*.

### Genome editing using the vector-based CRISPR/Cas9 system in other *Fusarium* species

**Figure 4:**
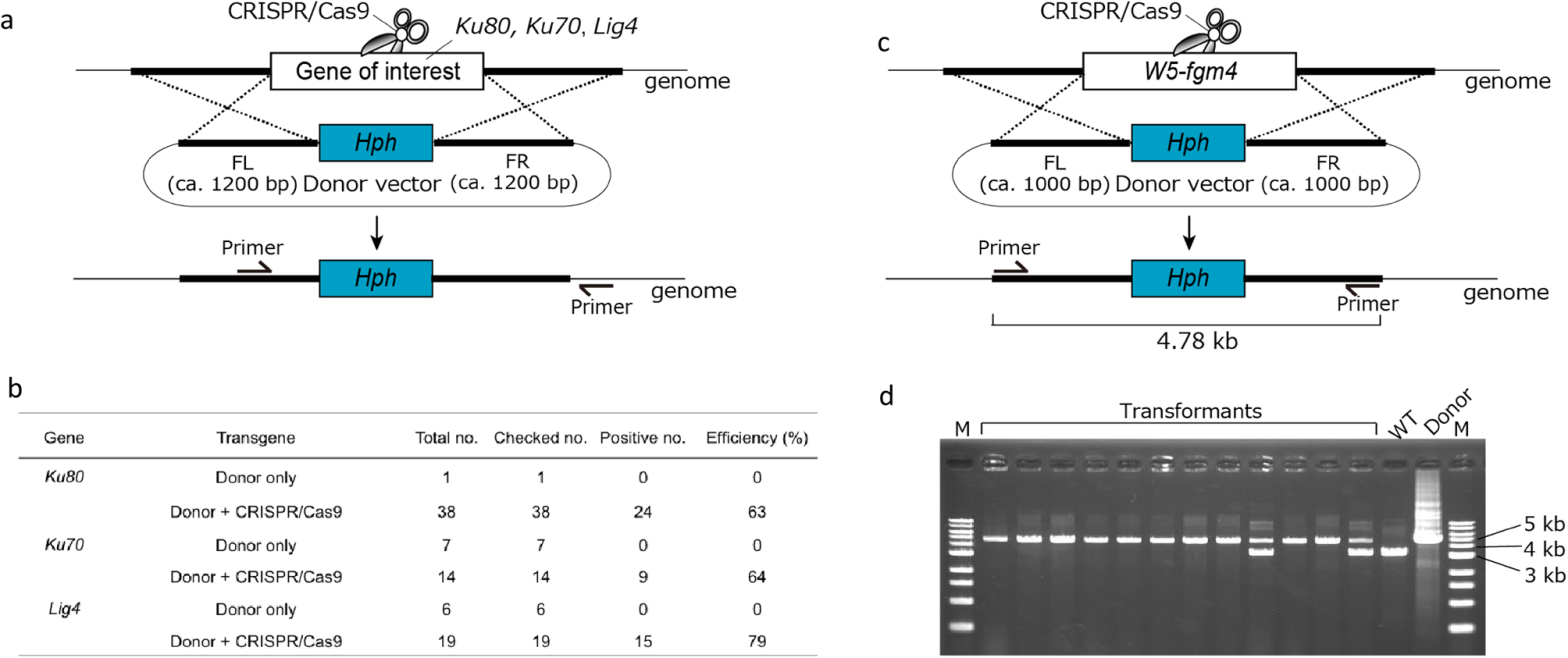
HR-mediated targeted gene knock-out using the developed CRISPR/Cas9 system in *Fusarium oxysporum* f. sp. *spinaciae* (*Fos*) Spin2SA and *F. commune* W5. (**a**) Schematic representation of HR-mediated targeted gene knock-outs in *Fos* Spin2SA. (**b**) The efficiencies of HR-mediated *Ku80*, *Ku70*, and *Lig4* knock-outs in *Fos* Spin2SA. (**c**) Schematic representation of HR-mediated *W5-fgm4* knock-out in *F. commune* W5. (**d**) PCR band-shift assay to detect the *fgm4-*like gene (*W5-fgm4*) knock-out mutants. Expected fragment sizes: wild type = 3200 bp and knock-out mutants = 4780 bp. M: 1 kb DNA ladder marker.

To investigate whether the developed CRISPR/Cas9 system can be used for other *Fusarium* species, we conducted genome editing in *Fos* isolate Spin2SA, the causal agent of Fusarium wilt of spinach, and *F. commune* isolate W5, a nonpathogenic biological control agent for *F. fujikuroi*. In these isolates, the transformation efficiency was lower than that of *Fol*. First, we evaluated the nuclease activity of the CRISPR/Cas9 vector by HR-mediated genome editing in *Fos* Spin2SA (Fig. 4a). The co-introduction with donor and CRISPR/Cas9 vectors increased the number of transformants, and the knock-out efficiencies of *Ku80*, *Ku70*, and *Lig4* were 63%, 64%, and 79%, respectively (Fig. 4b). Next, we employed the same strategy for *F. commune* W5 genome editing. In *F. graminearum*, the secondary metabolite biosynthesis cluster *fg3_54* has been identified as a virulence factor required for cell-to-cell invasion of wheat^27^. Interestingly, we identified an *fg3_53*-like gene cluster in the nonpathogenic *F. commune* W5, and an *fgm4-*like gene (*W5-fgm4*), a homolog of a pathway-specific bANK-like regulatory gene in *F. graminearum*^27^, was selected as the target gene (Fig. 4c). The introduction with only the donor vector could not produce any hygromycin B-resistant colonies, while 12 resistant colonies were obtained by the co-introduction with CRISPR/Cas9 and donor vectors. In addition, 10 out of 12 transformants (83.3%) were identified as disrupted mutants (Fig. 4d). These results indicated that the developed CRISPR/Cas9 system for *Fol* efficiently functioned in *Fos* and *F. commune* without any modifications.

## Discussion

Of the three CRISPR/Cas9 vectors evaluated in this study, the vector optimized for *P. oryzae* efficiently functioned in *Fol* also. Because the Tef promoter used for *Cas9* expression functions in both *P. oryzae* and *Fol*, the *P. oryzae* U6 promoter would also be effective in *Fol.* This result is consistent with a previous report describing the utility of the unmodified system for genome editing in the ATCC20542 filamentous fungal species^28^. On the other hand, the optimized vector, pCRISPR/Cas9-FoU6-FoNLS, dramatically increased the number of transformants and exhibited the highest editing efficiencies (100%) at the *Ku80*, *Ku70*, *Lig4*, and *ABHL* loci in *Fol*. These results indicated that the optimization process improved nuclease activity of the system, which would be capable of providing advanced genome editing in *Fusarium* species. In *Fol*, the efficient gene disruptions of *Ku80*, *Ku70*, and *Lig4* were also observed by introducing only the donor vector. As these genes are involved in the NHEJ repair, the donor vector may silence their expressions or functions during transformation; however, such events were not observed in the case of *Fos*.

We demonstrated highly efficient targeted gene knock-out, knock-in, and replacement strategies, including a part of AC regions. Short homology arm-mediated targeted gene disruption allows for the disruption of genes located in TE-enriched AC regions using short homologous sequences. Pathogenicity-related ACs have been analyzed using the chromosome-deficient strains that completely or partially lost ACs^5,29–31^. The developed CRISPR system would provide the additional strategy for efficient functional analysis of the individual genes on ACs. We also demonstrated the application of the CRISPR/Cas9 system for genome editing in *Fos* and *F. commune* without any modifications to the vector, suggesting that this system functions in a broad range of *Fusarium* species. We have confirmed the activity of this vector in *F. oxysporum* ff. spp. *conglutinans*, *pisi*, and *raphani* (data not shown). As the transient expression of CRISPR/Cas9 vector in fungal protoplasts not only improved the genome editing efficiency but also increased the number of transformants^14,15,23^, this strategy would be effective in *Fusarium* species with low protoplast isolation and/or transformation efficiencies.

We succeeded in efficient single-crossover-mediated targeted base editing and gene tagging in *Fol*. This strategy enables the introduction of perfectly designed mutations and one-step reporter gene knock-in at the desired genomic locus. Recently, a novel base editing technology, called prime editing (PE), was developed by using a nickase type Cas9 (Cas9^H840A^)-fused reverse transcriptase and a modified sgRNA (pegRNA)^32^. PE can introduce desired mutations at the nicked site without DSBs and donor DNAs but the expression of reverse transcriptase, insertion of the donor template at the 3’ end of pegRNA, and adequate PAM sequence are required. The donor vector for single crossover-mediated base editing can be constructed by one-step cloning, and any modifications of CRISPR/Cas9 are not needed. Further, this strategy can avoid the limitation of PAM sequence, though efficient HR induction is required. In *Fov,* homology-independent targeted integration (HITI) and homology-dependent recombination integration (HDRI) strategies have been established for endogenous gene tagging^18^. HITI enabled a large DNA fragment (~ 8 kb) integration including reporter genes but this strategy depends on a precise NHEJ repair at the cleaved DNA ends. Therefore, this process may cause frame shift mutations by incorrect ligations at the junction points of the products and cannot designate the direction of donor DNA fragments. HDRI depends on the double crossover-type or gene conversion-type HR. This strategy enabled the precise integration of donor DNAs at the target locus but it may be difficult to integrate large DNA fragments. Single crossover-mediated strategy enables an efficient and precise knock-in including large DNA integrations. Because single crossover-type HR was also detected in *F. oxysporum* ff. spp. *spinaceae* and *rahpani*, and *F. commune* (data not shown), this repair mechanism might be conserved in many filamentous fungi and would be applicable for a wide range of fungal genome editing.

Various CRISPR/Cas homologs and orthologs have been identified from a broad range of bacteria and archaea, and their application in flexible genome editing is now expanding^33^. In addition to genome editing, novel CRISPR systems and tools have been developed to perform gene regulation, epigenetic editing, and RNA editing^33^. One of the advantages of the vector-based CRISPR system is that it can be quickly modified and optimized for various types of CRISPR tools and organisms. Furthermore, the vector-based CRISPR system enables us to skip the procedures of in vitro sgRNA synthesis and Cas protein purification. Therefore, this system would be an additional tool for simple and effective genome editing in *Fusarium* species. We selected target sequences with low off-target effects and ensured efficient editing of all the tested genes. Further analysis, such as evaluation of more target loci, on-target effects, and multiple targets within a single gene, is required for further improvement of the CRISPR system and genome editing strategy in filamentous fungi. Our approach for optimization of the CRISPR/Cas9 system and the development of genome editing strategies would help progress other fungal genome editing studies. We anticipate that the development of genome editing strategies can expand the scope of a variety of basic and applied biological studies on *Fusarium* species and other filamentous fungi.

## Methods

### Fungal isolates, growth conditions, and DNA analysis

*Fol* isolate MAFF 103036 (race 1), *Fos* isolate Spin2SA, and *F. commune* isolate W5 were used in this study. These isolates were cultured on potato dextrose agar (PDA; 200 g/L potato broth, 0.5% (w/v) glucose, 1.5% (w/v) agar or Nissui Pharmaceutical Co.) or YG agar medium (0.5% (w/v) yeast extract, 2% (w/v) glucose, 1.5% agar) and stored in 25% (v/v) glycerol at − 80 °C. PCR and sequencing analyses were performed using standard procedures^8,15,35–37^. The primers and oligonucleotides used in this study are listed in Table S1.

### Optimization and construction of CRISPR/Cas9 vectors

The endogenous U6 promoter region of *Fol* with the *Esp*3 I site was synthesized and inserted into the *Not* I and *Sph* I sites of pCRISPR/Cas U6-1^23^, resulting in pCRISPR/Cas9-FoU6. The full sequence of the sgRNA expression cassette is shown in Fig. S4. The N-terminal Cas9 region attached to the NLS of histone H2B in *Fol* was created using fusion PCR. For the first PCR, the N-terminal Cas9 and NLS regions were amplified from pCRISPR/Cas9-FoU6 using an NLSCas9-1/-2 primer set and from the *Fol* genome using an NLSCas9-3/-4 primer set, respectively. The PCR products obtained from the first PCR were mixed and fused by a second PCR performed using an NLSCas9-1/-4 primer set. The N-terminal Cas9 region with NLS generated by fusion PCR was cloned between the *Asc* I and *Nde* I sites of pCRISPR/Cas9-FoU6, resulting in pCRISPR/Cas9-FoU6-FoNLS.

For the construction of CRISPR/Cas9 expression vectors targeting endogenous genes, each oligonucleotide set of Ku80-gRNA-1/Ku80-pogRNA-2, Ku80-gRNA-1/-2, Ku70-gRNA-1/Ku70-pogRNA-2, Ku70-gRNA-1/-2, Lig4-gRNA-1/Lig4-pogRNA-2, Lig4-gRNA-1/-2, ABHL-gRNA-1/ABHL-pogRNA-2, ABHL-gRNA-1/-2, SIX1-gRNA-1/-2, or Fgm4-gRNA-1/-2 was annealed according to the procedures of a previous report^15,23^. The annealed oligonucleotides were inserted into pCRISPR/Cas U6-1, pCRISPR/Cas9-FoU6 or pCRISPR/Cas9-FoU6-FoNLS via the Golden Gate cloning method according to a previous report^15,23,37^. CRISPR/Cas9 expression vectors for *Ku80*, *Ku70*, and *Lig4* were also used for *Fos* genome editing.

### Construction of donor vectors and DNA fragments

For the construction of the knock-out vector for *Ku80*, the flanking regions were amplified using the primer sets of TKu80-1/-2 and TKu80-3/4. The PCR product generated by the TKu80-1/-2 primer set was inserted between the *Sph* I and *Xho* I sites of pMK-dGFP^34^, resulting in pMK-Ku80-FL. The PCR product generated by the TKu80-3/4 primer set was inserted between the *Hin*d III and *Spe* I sites of pMK-Ku80-FL, which produced the donor vector for the knock-out of *Ku80* (pMK-Ku80-FLFR). For the construction of the knock-out vector for *Ku70*, the flanking regions were amplified using each primer set of TKu70-1/-2 or TKu70-3/-4. The PCR product generated by the TKu70-1/-2 primer set was inserted between the *Kpn* I and *Sph* I sites of pMK-dGFP (pMK-Ku70-FL). The PCR product generated by the TKu70-3/-4 primer set was inserted between the *Hin*d III and *Not* I sites of pMK-Ku70-FL, which produced the donor vector for the knock-out of *Ku70* (pMK-Ku70-FLFR). For the construction of the knock-out vector for *Lig4*, the flanking regions were amplified using each primer set of TLig4-1/-2 or TLig4-3/-4. The PCR product generated by the TLig4-1/-2 primer set was inserted between the *Kpn* I and *Sph* I sites of pMK-dGFP (pMK-Lig4-FL). The PCR product generated by the TLig4-3/-4 primer set was inserted between the *Sal* I and *Spe* I sites of pMK-Lig4-FL, which produced the donor vector for the knock-out of *Lig4* (pMK-Lig4-FLFR). For the construction of the knock-out vector for *ABHL*, the flanking regions were amplified using each primer set of TABHL1-1/-2 or TABHL-3/-4. The PCR product generated by the TABHL-1/-2 primer set was inserted between the *Kpn* I and *Xho* I sites of pMK-dGFP (pMK-ABHL-FL). The PCR product generated by the TABHL-3/-4 primer set was inserted between the *Hin*d III and *Sac* I sites of pMK-ABHL-FL, which produced in the donor vector for *ABHL* knock-out (pMK-ABHL-FLFR). For the construction of the knock-out vector for *SIX1*, the PCR products generated by each primer set of SIX1_P1/P2 or SIX1_P3/P4 were mixed with the *hph* cassette, amplified from pCSN43^40^ using a SIX1_pCSN_F/R primer set, and fused by PCR using a SIX1_P1/P4 primer set. The PCR product was attached to an adenine at the ends using a 10 × A-attachment mix (TOYOBO) according to the manufacturer’s protocol and cloned into pCR-XL-TOPO (Thermo Fisher Scientific), which produced in the donor vector for the knock-out of *SIX1* (pCSN43-SIX1-FLFR). For the construction of the knock-out vector for *W5-fgm4*, the PCR products generated by each primer set of fgm4_P1/P2 or fgm4_P3/P4 were mixed with the *hph* cassette, amplified from pCSN43 using a fgm4_pCSN43_F/R primer set, and fused by PCR using an Fgm4_P1/P4 primer set. The PCR product was attached to adenine at the ends by the 10 × A-attachment mix and cloned into pCR-XL-TOPO, which produced in the donor vector for the knock-out of *W5-fgm4* (pCSN43-*W5-fgm4*-FLFR).

For the construction of the donor DNA fragments with truncated short homology arms, fragments with 1000, 750, 500, 250, 100, and 50 bp homology arms for *Ku80* were amplified from pMK-Ku80-FLFR using Ku80-1000-1/-2, Ku80-750–1/-2, Ku80-500-1/-2, Ku80-250-1/-2, Ku80-100-1/-2, and Ku80-50-1/-2, respectively. Similarly, donor fragments with 1000, 750, 500, 250, 100, and 50 bp homology arms for *Lig4* were amplified from pMK-Lig4-FLFR using the primer sets Lig4-1000-1/-2, Lig4-750-1/-2, Lig4-500-1/-2, Lig4-250-1/-2, Lig4-100-1/-2, or Lig4-50-1/-2, respectively. Donor fragments with 1000, 750, 500, 250, 100, and 50 bp homology arms for *ABHL* were amplified from pMK-ABHL-FLFR using the primer sets of ABHL-1000-1/-2, ABHL-750-1/-2, ABHL-500-1/-2, ABHL-250-1/-2, ABHL-100-1/-2, and ABHL-50-1/-2, respectively.

For the construction of the donor vector of the single crossover-mediated *Ku80* base editing, the *Ku80* gene region comprising point mutations was generated by fusion PCR. For the first PCR, the *Ku80* gene regions were amplified from the genome of *Fol* using each primer set of SKu80-1/-2 or SKu80-3/-4. The PCR products obtained from the first PCR were mixed and fused by the second PCR using the SKu80-1/-4 primer set. The *Ku80* gene region with point mutations obtained by fusion PCR was inserted between the *HindIII* and *SpeI* sites of pMK-dGFP, which produced in pMK-SKu80.

For the construction of the donor vector of the single crossover-mediated *Ku80* gene tagging, GFP-fused *Ku80* gene was generated by fusion PCR. For the first PCR, the *Ku80* gene regions with silent mutations, with deleted start and stop codons, were amplified from pMK-SKu80 using SKu80-1/KKu80-2. The amplified *Ku80* gene region was inserted between the *Hin*d III and *Spe* I sites of pMK-dGFP (pMK-KKu80). The *GFP* gene amplified from pMK412^35^ using a KK80-3/KK80-4 primer set was cloned between the *Spe* I and *Sac* I sites of pMK-KKu80, resulting in pMK-KKu80-GFP.

### Protoplasts preparation and transformation of *Fusarium* species

Protoplasts of *F. commune* were prepared as previously described^38^. In the case of *Fol* and *Fos*, protoplasts were isolated similar to previous reports^35,39^ with some modifications. *Fol* or *Fos* was cultured in 30 mL of YG liquid medium at 28 °C for 1 day at 180 rpm. One milliliter of the culture was transferred into 100 mL fresh YG medium and incubated at 28 °C and 180 rpm overnight. The mycelia were collected by centrifugation at 12,000 rpm for 10 min and washed twice with 1.2 M MgSO_4_. In the case of *Fol*, the mycelia were treated with a mixture of 20 mg/mL Yatalase (Takara bio, Japan), 20 mg/mL lysing enzyme (Sigma, USA), and 20 mg/mL cellulase (Yakult Pharmaceutical Industry, Japan) in 1.2 M MgSO_4_ for 3–4 h. In the case of *Fos*, the mycelia were treated with a mixture of 40 mg/mL Yatalase, 40 mg/mL Driserase (ASKA Animal Health, Japan), 40 mg/mL lysing enzyme (Sigma), and 40 mg/mL cellulase in 1.2 M MgSO_4_ for 16 h. The fungal protoplasts were collected and washed twice with STC (1.2 M sorbitol, 50 mM CaCl_2_, and 10 mM Tris–HCl pH 7.5). Then, the protoplasts were diluted to a concentration of 5 × 10^6^/mL with STC buffer.

Approximately 2.5 µg each of CRISPR/Cas9 and donor vectors were mixed (total volume 5 µL) and added to 50 µL of the protoplast suspension. Approximately 2.5 µg of donor vectors (total volume 5 µL) were used as a control (donor only). In the case of the donor DNA fragments obtained by the truncation of homology arms, approximately 1 µg of CRISPR/Cas9 vectors and 500 ng of donor DNA fragments were mixed (total volume 5 µL) and added to 50 µL of the protoplast suspension. Approximately 500 ng of the donor fragments (total volume 5 µL) were used as a control (donor only). For the disruption of *SIX1* and *W5-fgm4*, approximately 15 µg of each donor vector and CRISPR/Cas9 vector were mixed (total volume 15 µL) and added to 150 µL of the protoplast suspensions. Approximately 15 µg of donor vectors (total volume 15 µL) were used as a control (donor only). The transformants were selected and maintained on YG solid medium containing hygromycin B (100 µg/mL). The primers used for the PCR analysis to check the disrupted mutants are listed in Table S1. To check the *SIX1* and *W5-fgm4* disruptions, the primer sets of SIX1_P1/P4 and fgm4_P1/P4 were used, respectively. To check the sequences obtained by single crossover-mediated genome editing, a Ku80-check-1/Ku80-seq-2 primer set was used for the PCR, and Ku80-seq-2 was also used for the sequencing analysis.

## Supplementary Information

Supplementary Information 1.

Supplementary Information 2.

## Data Availability

The datasets generated and/or analyzed during the current study are available in the NCBI (https://www.ncbi.nlm.nih.gov) under the following accession numbers: *Ku80*, FOXG_02215; *Ku70*, FOXG_06386; *Lig4*, FOXG_11480; *ABHL*, FOXG_14298; *SIX1, FOXG_16418*; *W5 fgm4*, LC708252; Fos genome, GCA_013347345.1. All data generated or analyzed during this study are available from the corresponding author upon reasonable request.
