# Supplementary material for: Genome editing using a versatile vector-based CRISPR/Cas9 system in Fusarium species
Source: Sci Rep. 2022 Sep 28;12:16243. doi: 10.1038/s41598-022-20697-4 (PMC9519947; doi:10.1038/s41598-022-20697-4)
Supplement: Supplementary file 1 — Supplementary Information 1. [file 41598_2022_20697_MOESM1_ESM.docx]

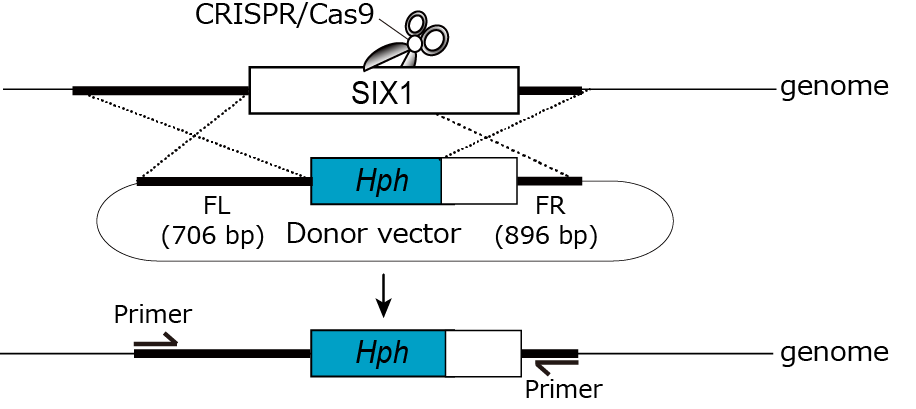


Figure S1. Homologous recombination (HR)-mediated *SIX1* knock-out using optimized CRISPR/Cas9 system in *Fusarium oxysporum* f. sp. *lycopersici* (*Fol*). Schematic representation of HR-mediated targeted gene knock-out with CRISPR/Cas9 system. FL: flanking region of left side, FR: flanking region of right side, *Hph*: hygromycinB phosphotransferase gene cassette.

b

a


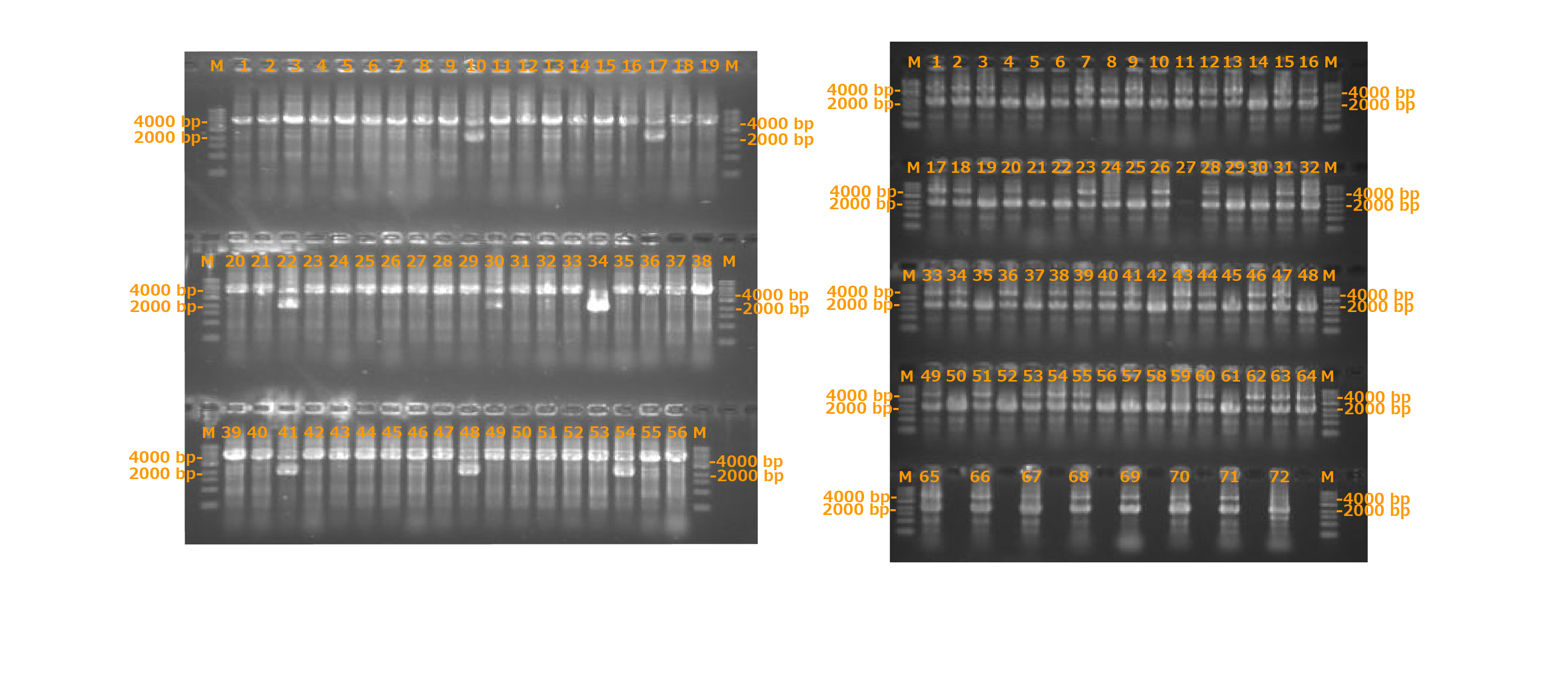


Figure S2. HR-mediated *SIX1* knock-out using CIRPSR/Cas9 system

(a) PCR band-shift assay of the transformants obtained by co-introduction with the CRISPR and donor vectors. (b) PCR band-shift assay of the transformants obtained by co-introduction with the only donor vector. Expected fragment sizes: wild type=2,009 bp and knock-out mutants=4,326 bp. M: 1 kb DNA ladder marker.

a


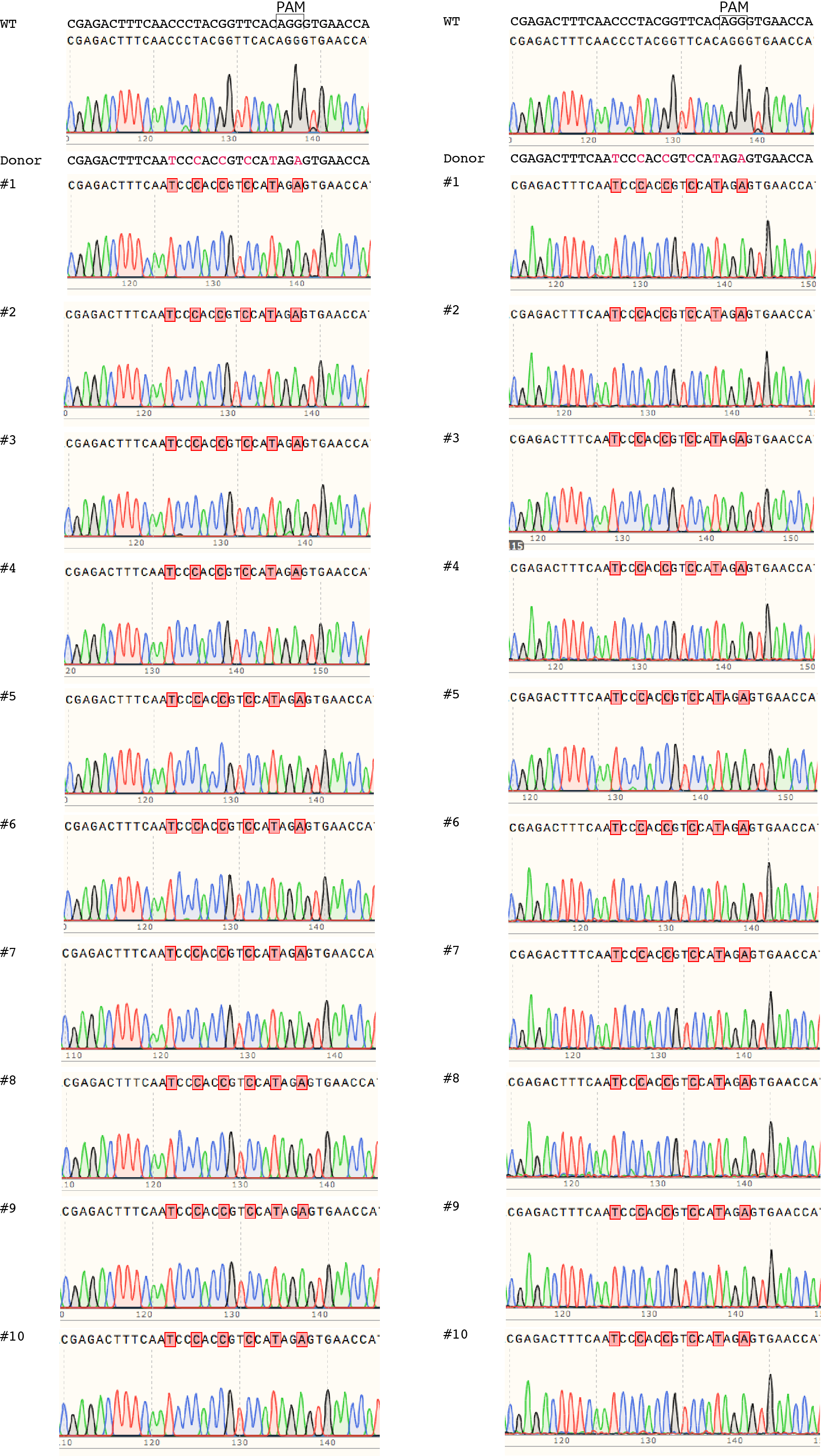


b

Figure S3. Sequencing chromas data around the CRISPR/Cas9 target site in the transformants obtained by single crossover mediated base editing and gene tagging. (a) All of the chromas data obtained by *Ku80* base editing. (b) All of the chromas data obtained by *Ku80* tagging with *GFP*.

gccagatatattggaccgagtcggccgtgctggggatctctaacgctgaaaagcgacaagaacactttccagcatgaaaacagacaaaagaactctctccagagccttttgattgatacaagggagcgttggtgaggaccatcagaggctgacgatgccactgtgagcagggacccctgtcataaaggctggcaaaacaagggtatataggttgtaattgcaaactctctctcgggagacgggatccgcatgccgtctccgttttagagctagaaatagcaagttaaaataaggctagtccgttatcaacttgaaaaagtggcaccgagtcggtgcttttttt

Figure S4. Nucleotide sequence of the sgRNA expression cassette. Red letters indicate U6 promoter. Blue letters indicate Esp3I sites. Green letters indicate sgRNA scaffold.
